# Age-related differences in temporal binding and the influence of action body parts

**DOI:** 10.1177/20416695231208547

**Published:** 2023-10-30

**Authors:** Yoshitaka Fujii, Naoki Kuroda, Ryo Teraoka, Shinya Harada, Wataru Teramoto

**Affiliations:** 623388 College of Comprehensive Psychology, Ritsumeikan University, Ibaraki-shi, Osaka, Japan; 624719 Graduate School of Humanities and Social Sciences, Kumamoto University, Chuo-ku, Kumamoto, Japan; Graduate School of Humanities and Social Sciences, Kumamoto University, Chuo-ku, Kumamoto, Japan; 624719 Graduate School of Social and Cultural Sciences, Kumamoto University, Chuo-ku, Kumamoto, Japan; Graduate School of Engineering, Muroran Institute of Technology, Muroran, Hokkaido, Japan; Graduate School of Humanities and Social Sciences, Kumamoto University, Chuo-ku, Kumamoto, Japan; Graduate School of Humanities and Social Sciences, Kumamoto University, Chuo-ku, Kumamoto, Japan; Graduate School of Humanities and Social Sciences, Kumamoto University, Chuo-ku, Kumamoto, Japan

**Keywords:** temporal binding, intentional binding, causal binding, perception of older age, time perception, sense of agency, voluntary action

## Abstract

If voluntary action is followed by an effect with a short time delay, the time interval between action and effect is often perceived to be shorter than it actually is. This perceptual time compression is termed intentional binding or temporal binding. We investigated age-related changes in adulthood considering temporal binding and its dependence on action body parts (i.e., hand vs. foot). This experiment included 17 young adults (mean age: 21.71 ± 3.14 years) and 27 older adults (mean age: 74.41 ± 3.38 years). Participants performed a button press task using their index fingers (hand condition) or toes (foot condition). The results showed that older participants exhibited a strong time compression comparable to young participants in the voluntary condition. Older participants also showed a strong time compression in involuntary action, which was induced by a mechanical device, differently from young participants. In line with previous research, the present age-related differences in time compression considering involuntary action suggest that causal belief significantly influences event perception rather than the associated intention of action or sensory afferents. The present results also suggest that the nature of action body parts has no significant influence on temporal binding, independent of age group.

Distinguishing between the consequences of one's own actions and those of others is fundamental throughout life in order to interact appropriately with the environment. Haggard et al. (2002) reported an interesting phenomenon closely related to such action–effect matching: a perceived time interval between action and following sound effect with a short delay can be influenced by action intention. The authors measured the perceptual time interval between an action and the following sound with a 250 ms delay using the Libet clock method (Libet et al., 1983), where participants answered the rotating clock hand position when an event occurred. They found that the perceived time interval changed depending on whether the action was performed voluntarily or involuntarily. Specifically, voluntary action induced the perceived time compression between the action and its effect compared with the actual time interval, while involuntary action (muscle twitch triggered by transcranial magnetic stimulation (TMS) of the motor cortex related to finger movement) did not induce such time compression. Haggard et al. (2002) suggested the existence of a distinct brain module that binds intentional action and its effect. This perceptual time compression during voluntary action is termed intentional binding or temporal binding (TB) and has repeatedly been confirmed in several studies (Haggard, 2017; Haggard & Chambon, 2012; Haggard et al., 2002; Moore & Obhi, 2012).

Despite a considerable number of studies reporting the phenomenon, the underlying mechanism for TB remains unclear. Haggard et al. (2002) explained that TB arises from prediction mechanisms based on sensory-motor integration between action and following effect and argued for a close association with sense of agency (Haggard, 2017). Moore et al. (2010) revealed that TB was reduced when the presupplementary motor area, “a key structure for preparation and initiation of voluntary action,” was disturbed by theta-burst stimulation of TMS. Studies also suggest that TB reflects a motor prediction mechanism based on a forward model of action (Engbert & Wohlschläger, 2007; Tsakiris & Haggard, 2003). Contrarily, more recent studies have suggested the involvement of postdictive or retrospective inference mechanisms (Buehner, 2012; Buehner & Humphreys, 2009; Kawabe et al., 2013; Klaffehn et al., 2021; Lorimer et al., 2020; Moore et al., 2009; Wolpe et al., 2013). Buehner (2012) has developed a button press machine that can establish an unambiguous causal relationship between the machine's button press and the following effect. Additionally, it showed that the machine's action induced perceived time compression even though participants made no action. Some studies also substantiated the importance of causality rather than action intention (Buehner & Humphreys, 2009; Hoerl et al., 2020; Lorimer et al., 2020; Suzuki et al., 2019). Studies have shown that multisensory perceptual grouping or cue integration processes underlie this action–effect binding (Kawabe et al., 2013; Klaffehn et al., 2021; Lush et al., 2019; Yamamoto, 2020). Therefore, both mechanisms more or less contribute to TB (see Haggard, 2017; Moore & Haggard, 2008 for a review).

It is well known that physical abilities for actions such as muscle strength and body flexibility decrease with age. Sensory and cognitive abilities can also decrease with age (Park & Reuter-Lorenz, 2009; Seidler et al., 2010; Owsley, 2011). Because the binding between action and effect is assumingly established by sensory, motor, and cognitive functions, the binding is also possible to face age-related changes. Indeed, studies reported age-related changes in sensory-motor prediction (Wolpe et al., 2016), sense of agency (Moore, 2016), causal inferences (Mutter & Plumlee, 2009; Mutter et al., 2007), and multisensory integration mechanisms (Jones & Noppeney, 2021). Therefore, TB is likely to change with aging. However, little is known about how aging influences TB. Cavazzana et al. (2017) measured TB in children (aged 8–11 years) as well as young (aged 22–30 years) and older adults (aged 66–76 years). They employed a unique method instead of the Libet clock paradigm, which has been widely used since Haggard et al.'s study (2002) because the authors considered that the cognitive ability to read a clock is not fully developed in children. Thus, a sequence of single alphabets was rapidly presented one after another with short interonset latency (150 ms) between the alphabets. Participants were required to remember the alphabet when a specific event occurred while they were observing the sequence. Perceptual time shift was calculated as the difference in the presentation timing of alphabets where an actual event occurred and the respective response. Cavazzana et al. (2017) concluded that TB for children and older adults was weaker than for young adults because of difficulties in attentional control. That is, children and older adults find it difficult to switch their attention between events and tend to focus on their voluntary action. Hence, the perceptual time shift of voluntary action is hardly induced. However, Lorimer et al. (2020) suggested the possibility that Cavazzana's method placed more attentional load than other paradigms, resulting in reduced TB for children and older adults. Indeed, Lorimer et al. (2020) showed that the TB for children was comparable to that for young adults when they used a method that induced only low cognitive load. Thus, the findings of Cavazzana et al. (2017) regarding older adults might be due to their unique method. We aimed to further investigate older adults’ TB using the conventional Libet clock paradigm.

In addition to hand actions, foot actions are especially important for older adults because of the risk of falls; unexpected falls are a major cause of developing serious disabilities and becoming bedridden among those in this age group. When climbing stairs, dodging, simply walking, and in various risky situations, timing and foot action are important factors. Given that TB is the most typical phenomenon of time perception with action, investigation of the age-related changes of TB induced by foot action may provide hints to reducing the risk of falling in older adults. Therefore, we also investigated TB induced not only by hand but also by foot action, considering both young and older adults. There are many differences between hands and feet and their respective actions. For example, the hand can grasp and move accurately; the foot can support the weight of the whole body and is powerful enough to walk and jump; the neurotransmission length is also different. Therefore, it can be that foot action induces TB differently from hand action. Especially considering older adults, Janssen et al. (2000) reported that the muscle mass of the lower limbs decreases more rapidly than that of the upper limbs in association with aging. Therefore, TB is possible to show different relationships with aging depending on whether hand or foot actions are considered.

## Method

### Participants

The experiment included two participant groups: young and older adults. Young adults were 17 students from Kumamoto University (11 men and 6 women; mean age: 21.71 ± 3.14 years; all right-handed) who were naïve to the study procedure. Older adults were 27 individuals (14 men and 13 women; mean age: 74.41 ± 3.38 years; 26 right-handed and 1 ambidextrous) recruited via a local subsidiary of the National Human Resource Center for Seniors. The dominant hand was assessed with Chapman's handedness questionnaire (Chapman & Chapman, 1987). The sample size was determined a priori considering previous TB studies and our pilot experiments. For example, in Cavazzana et al. (2017), a final sample of 20 young adults and 18 older adults was included. Based on this and similar studies, we considered a sample size of 20 young adults as reasonable. For the older adult group, contrastingly, we recruited all the older adults available in our participant pool at the time (29 individuals) because we expected a higher dropout rate owing to deficits in cognitive or sensory functions. However, the actual dropout was only two participants. Although the number of older participants was larger than that of young participants, we decided not to decrease the number of older participants. According to a power analysis conducted in G*power 3.1.9.6 (Faul et al., 2007, 2009), 14 participants were required in each group for a statistical power of 0.8 for a between–within interaction (a 2 × 3 design), assuming a medium effect size of *f* = 0.25 and α = .05. Participants in the older group performed a hearing test and the Mini-Mental State Examination (MMSE; Folstein et al., 1975). The hearing test confirmed that all participants could hear 50 dB sound pressure level (SPL) pure tone in at least one ear, both in 1 kHz and 2 kHz sound frequencies, and that the hearing level was high enough to conduct the experiment. All older participants scored 27 or more on the MMSE, indicating no cognitive impairment (mean years of schooling = 13.56 ± 1.97). All participants had normal or corrected-to-normal vision. Of note, some of the older participants had mild eye diseases such as mild senile cataracts and mild age-related macular degeneration.

This study was conducted in accordance with the principles of the Declaration of Helsinki, and all its amendments were approved by the Ethics Committee of the Graduate School of Social and Cultural Sciences, Kumamoto University (2020-No. 51). Participants provided written informed consent prior to participating in this study.

### Conditions

To measure the influence of action intention on the perceived time distance between action and target sound, there were two groups of conditions for each body part: event sequence conditions and single-event baseline conditions. All event sequence conditions consisted of one of three preceding events and the following target sound. The preceding events were (a) voluntary action, (b) involuntary action, or (c) control sound (sound play with no action). The control sound condition was to investigate a simple perceptual time interval with no action as a control. In each of these three sequences, participants were required to report the event timing of either the preceding event or the following target sound. Thus, six conditions were performed in total (three-event sequences × two response targets).

There were four single-event baseline conditions: (a) voluntary button press, (b) involuntary button press, (c) control sound play, and (d) target sound play. Participants were required to report the event timing of the button press except for the single sound play condition. These baseline conditions were used to eliminate the influence of a single event from those of the event sequence conditions (see the Analysis section for further details).

In sum, the total number of trial conditions was 10 (six sequence conditions and four single baseline conditions) for each body part. Each condition was conducted in a different block, each of which included 5 practice trials and 20 experimental trials for young adults, and 5 practice trials and 10 experimental trials for older adults. A difference in the number of experimental trials was implemented to reduce load in older participants. The order of blocks was randomized for each participant. Participants were allowed to rest between the blocks. Responses to the practice trials were not recorded. The hand session always preceded the foot session.

### Apparatus and Stimuli

**Figure 1.**
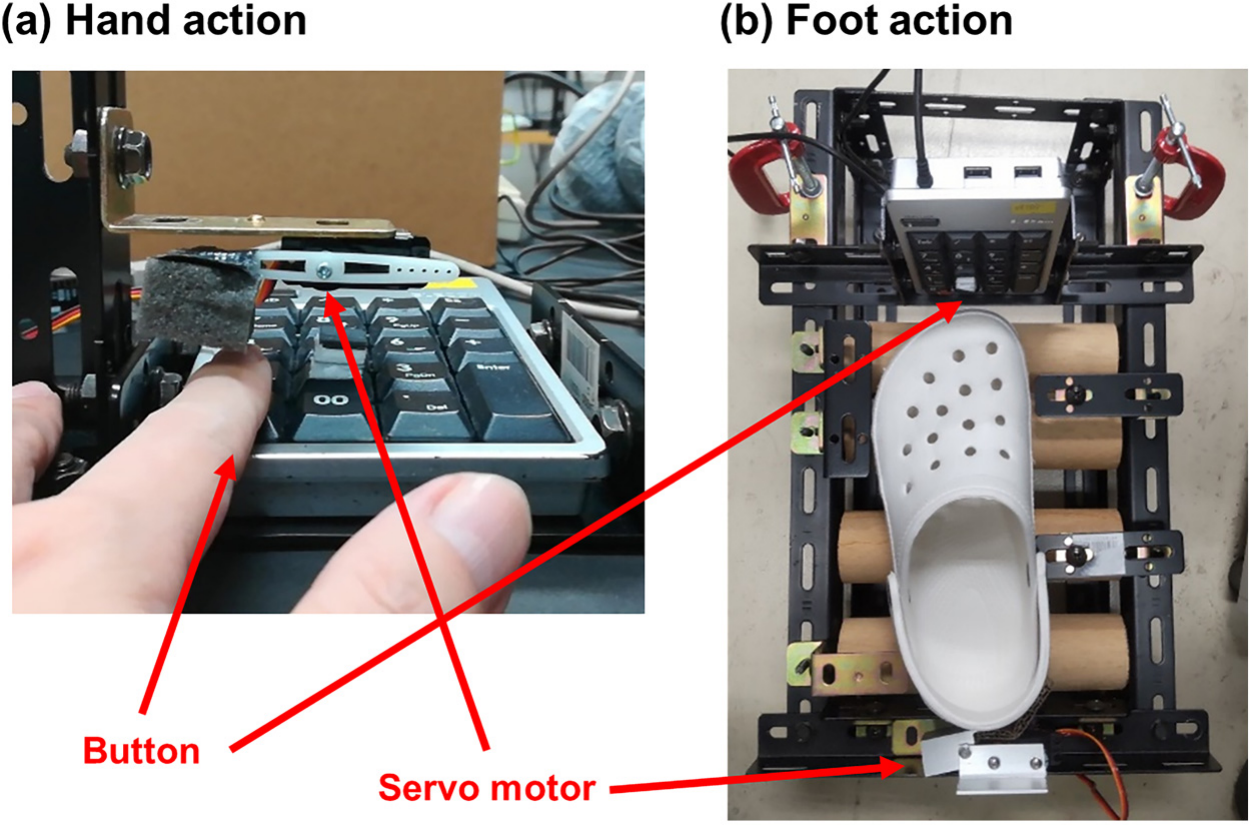
(a) Frontal view of the button press device for hand action. For the involuntary button press, the white lever (center) was attached to the servo motor, which revolved counter-clockwise so that the left index finger under the foam cube was forced downward. (b) Top view of button press device for foot action. Participants wore the white plastic shows (at the center) and put on rollers. For involuntary button press, the metal lever (bottom) was attached to the servo motor, which revolves clockwise so that the left foot in the plastic show was forced forward (upward in the figure), and the toes pushed the button.

The apparatus consisted of an operation computer, a liquid crystal display touch display, a noise sound player, headphones, and button press devices (a keypad attached to a mechanical device for involuntary action). The experiment was controlled by Octave (version 4.1.1, GNU Project) with the Psychtoolbox-3 (Brainard, 1997; Kleiner et al., 2007; Pelli, 1997) extension on the operation computer (Dospara, GALLERIA GCR2070RGF-QC; Ubuntu Linux, version 1910, Canonical UK Ltd/Ubuntu Project). The touch display (Bosstouch PM156; display size: 34.5 cm × 19.5 cm; resolution: 1920 pixel × 1080 pixel; refresh rate: 60 Hz) was used for the presentation of visual stimuli and acquisition of participants’ responses. The touch display was placed horizontally on a desk, and the participants looked down at the touch display. The center of the display was approximately 30 cm in front of the observer. The viewing distance was approximately 50 cm. A keypad was used to detect voluntary and involuntary button presses. For the hand session, participants placed their left index finger on the keypad (Figure 1a). A lever connected to a servo motor was set above the left index finger and applied force to the finger for the involuntary key press. For foot action, participants wore plastic shoes, and their left foot was put on wooden rollers, which allowed smooth back-and-forth movements (Figure 1b). The keypad was placed in front of their left toes vertically. For the voluntary button press, participants moved the foot forward on the rollers, and the toes of the plastic shoe pressed the button. For involuntary foot action, a servo motor behind the heel applied force to the foot such that the foot moved forward to press the button. This device was under the desk on the floor. The participant's right foot was on a footstall whose height was adjusted to the height of the device for the left foot to keep a balanced posture. The servo motor was controlled via a USB-connected microcomputer Arduino (Arduino Uno, Arduino Holding).

A clock-shaped stimulus similar to that used in Haggard et al. (2002) was presented. The clock stimulus consisted of a circle frame, a clock hand, a clock scale, and the surrounding digits; all of them were white, drawn on a black background. The clock scale was drawn every 30° from the top like in a common analog clock. Moreover, the clock digits were 0 at the top and increased by 5 clockwise (i.e., 0, 5, 10, …, 55). In older adults, larger clock digits were to help recognition. The clock hand rotated clockwise with a period of 2560 ms. The starting position of the clock hand was randomized. The control sound (substitute event for button press) and following target sound (with 250 ms interonset latency following the button press) were 2 kHz and 1 kHz pure tones (sampling frequency: 44.1 kHz; 71 dB SPL) with a duration of 100 ms, respectively. The sound stimuli were presented via headphones (Sony MDR-CD900ST, Japan). Pink noise was also presented to mask the motor noise of the button press device. We confirmed that the control sound and target sound were audible without difficulty under the masking noise.

### Procedure

The experiment was performed in a room under fluorescent lighting. The participants sat in front of a table and placed either their hand or foot on the button press device. They were instructed to keep their hand or foot in place during the session to hold their posture constant even if button press action was not required in the condition. Participants were allowed to move their heads and eyes. At the beginning of each trial, the instructions were presented on the touch display, and participants confirmed the upcoming event(s) and the response target. When the participants touched a start button on the display using their right hand, the instructions disappeared, and the clock stimulus appeared immediately. The participants observed the clock and remembered the position of the clock hand when the response target event occurred. In the voluntary action conditions, participants pressed the button anytime they wanted (even if just after the start of the trial). However, they were instructed to avoid predetermining the time to press because a predetermined time could bias their response about the positions. In the involuntary action conditions and sound play conditions, the first event occurred randomly between 1000 and 2000 ms after the appearance of the clock stimulus. After the last event, the clock presented 1000–2000 ms randomly and disappeared. Another clock for response appeared soon after a short blank. The response clock was the same, except the clock hand had stopped. However, the participants could move the clock hand by touching the display. They reproduced the perceived clock hand position when the response target event occurred and pressed the confirmation button on the display. The final position when a button was touched was recorded as the response in that trial.

### Assessment of Sense of Agency (Supplemental Session)

We measured the strength of intention and agency of the button press for several older adults who could participate in a supplemental session on a different day after the main sessions. Imaizumi and Tanno (2019) reported intraindividual correlations, as well as interindividual correlations, between the explicit sense of agency and TB, while Schwarz et al. (2019) suggested that the relationship between the explicit agency of action and TB is more complex. Thus, in each trial, participants performed a voluntary or involuntary action as a preceding event while observing the clock stimuli, and a sound stimulus followed with 250 ms interonset latency, as in the event sequence conditions. After the stimulus presentation, the participants were required to estimate the subjective strength of intention or agency of action with a visual analog scale on the touch display. Specifically, for the intention estimation, the participants were asked to estimate how much intention they felt when they moved their finger. The left end of the visual analog scale corresponded to “My finger (foot) moved without my intention at all,” and the right end corresponded to “My finger (foot) moved completely with my intention.” As for the agency estimation, the participants were asked to estimate how much they felt the button press was performed by their own action or machine action. The left end of the visual analog scale corresponded to “I am the one who pressed the button,” and the right end corresponded to “The machine is the one who pressed the button.” The participants answered the intention strength question before the agency strength question (we did not counterbalance the order to avoid confusion). Voluntary and involuntary actions were conducted in separate blocks. Each block included two practice trials and five experimental trials.

The visual analog scale consisted of a white horizontal line (15 cm) and short vertical lines at both ends of the horizontal line. A red vertical line was also drawn on the horizontal line. Participants were asked to move the red line with their touch control to answer their subjective strength of intention or agency. The percentage of length between the left vertical line and the red vertical line against the total length of the horizontal line was recorded as a score of response. Specifically, the left end, the center, and the right end corresponded to 0, 50, and 100 scores, respectively. The initial position of the red line for the response was randomized. Instructions to provide answers were also given above the scale.

### Analysis

Perceptual time shift (Δ*T* in ms) was calculated with the rotational period (*T*_0_: 2560 ms per round) and the difference of the clock hand angle (Δ*θ* in degree) between the time point when the participants responded and when the actual event occurred; that is, $\Delta T=\frac{T_{0}}{360}\cdot \Delta \theta$ . Positive time-shift values indicated that perception was delayed compared with the actual event.

The median of the time shifts across 20 (young participants) or 10 (older participants) repetitions of each condition was calculated as a representative value for each condition. The reason for using the median instead of the mean was to reduce the influence of outliers. To remove the single-event bias, which depended on a single event alone, the time shift of single baseline conditions was subtracted from the corresponding time shift in sequence conditions, that is, the time shift of action (action binding) was calculated as the subtraction of time shifts between each event sequence condition (response target was preceding event) and corresponding baseline condition, and the time shift of the following target sound (effect binding) was calculated as the subtraction of time shifts between event sequence condition (response target was the target sound) and the baseline condition of target sound play. Positive values indicated that event perception of the sequence condition was delayed compared to the single condition. Finally, perceived time distance (time compression) was calculated as the total sequence effect of the action and the target sound (i.e., action binding–effect binding). The positive values of time compression indicated a shorter perceptual time distance between the button press and target sound compared to the actual time distance. Because the hand and foot actions were not identical with different devices, the following analyses were performed by considering the hand and foot data independently.

Statistical analyses were performed using R software (version 4.0.5, https://www.R-project.org/). The significance level was set at *p* < .05 for all tests and analyses. Nonparametric analyses were applied because Shapiro–Wilk tests indicated a violation of the normal distribution assumption for part of the data (*p* < .05), and Levene's tests indicated a violation of equality of variance for some data (*p* < .05). The effect of the participant group was tested on each preceding event condition using the Brunner–Munzel (BM) test with the Bonferroni correction (corrected significance level: .017). We also performed the Friedman test to investigate the effect of the preceding event. The Nemenyi tests were used for the following multiple comparisons. The multiple comparisons between the preceding event conditions were performed not only when the effect was significant (*p* < .05) but also when it was marginally significant (.05 < *p* < .10) because the influence of the preceding event was a main interest of the experiment. These analyses were performed on each of the time compression, action binding, and effect binding data. The BM test statistic *B *(LaBone et al., 2013) was converted to a positive value. The probability of superiority (*PS*; 0.5 < *PS* < 1.0) was calculated as an effect size index when available (Grissom & Kim, 2012).

The responses of visual analog scales in the supplemental session were translated to the strength of intention/agency: 0 (no intention/agency) to 100 scores (the maximum intention/agency). The average of the strength scores across five repetitions of each condition was calculated as a representative value for each condition. Shapiro–Wilk tests indicated a violation of the normal distribution assumption in both body parts (*p* < .05). A Spearman's rank correlation coefficient analysis was performed to investigate whether the time compression of each participant was associated with their feeling of intention or agency of action. Note that the time compression data of only the older adults who participated in this experiment were submitted to this analysis.

## Results and Discussion

### Hand

**Figure 2.**
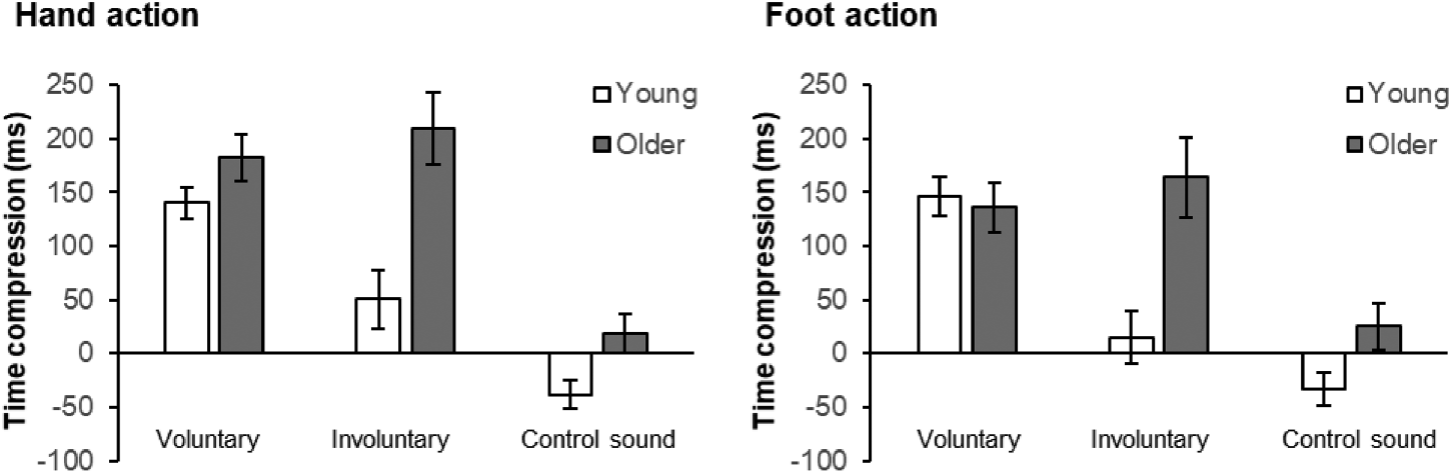
Results of time compression induced by hand action (left) and by foot action (right). The white bars and the gray bars indicate the results of young and older participants, respectively. Positive and negative values indicate shorter and longer perceived time distance between the preceding event and the following sound events than the actual time distance, respectively. The error bars indicate standard errors.

**Figure 3.**
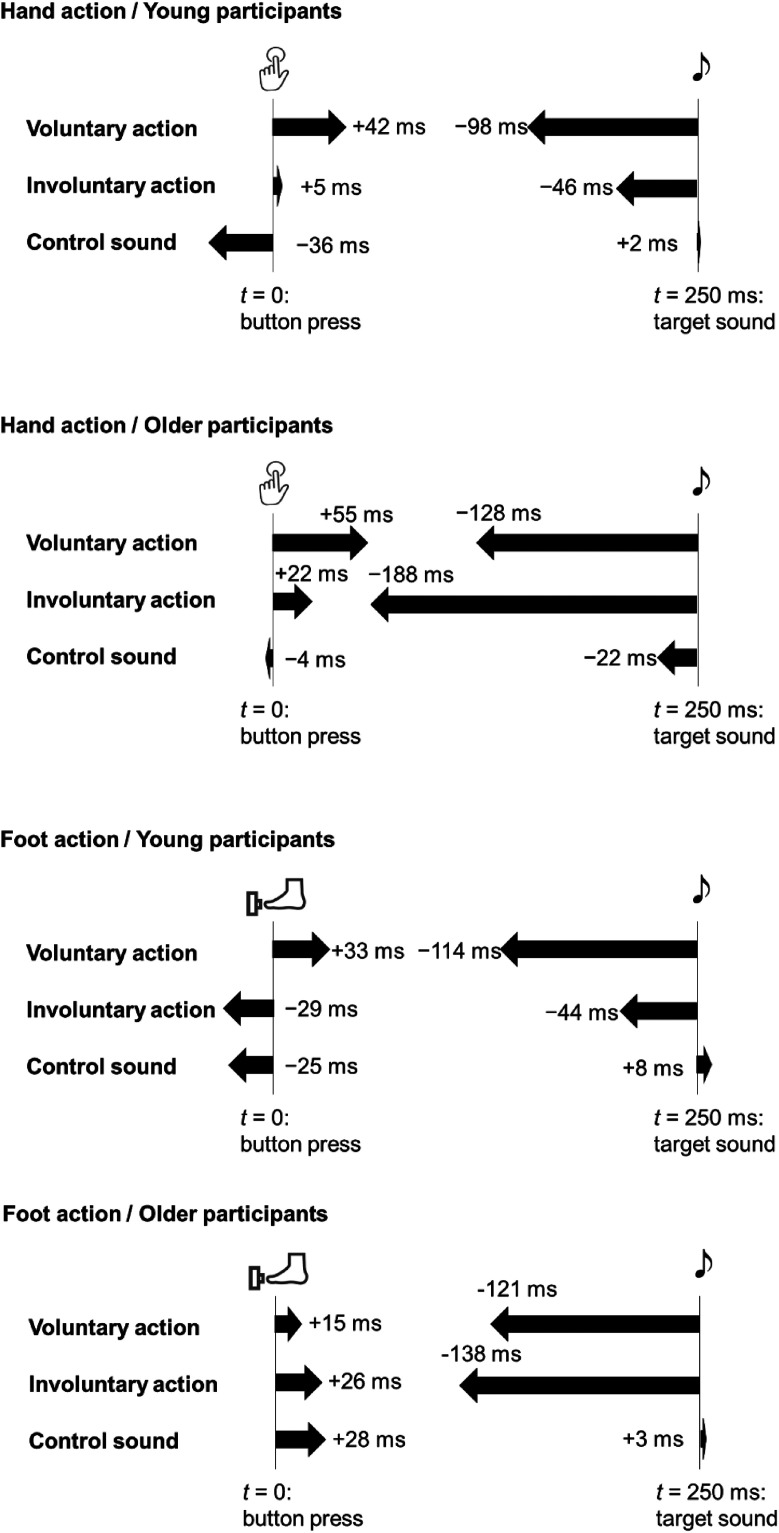
Overview of the results of corrected time shifts. The arrows and values represent the quantity of action bindings and effect bindings.

***Time compression.*** Figure 2(left) illustrates the results of time compression. Supplemental Table S1 shows the time shift of each condition, and Figure 3 (top two panels) illustrates an overview of each time shift after the correction with a single event condition. The BM tests revealed that the time compression was stronger in older than young participants in the involuntary (*B* = 3.85, *df* = 41.74, *p* < .001, *PS* = .780) and control sound conditions (*B* = 2.52, *df* = 40.05, *p* = .016, *PS* = .702), while there was no difference in the voluntary condition (*B* = 1.48, *df* = 37.30, *p* = .148, *PS* = .625). As for the effect of the preceding event, it was significant in both young and older adults (young: χ^2^(2) = 24.82, *p*_adjusted_* *< .001; older: χ^2^(2) = 24.52, *p*_adjusted_* *< .001). The multiple comparisons revealed significant differences in all pairs (*ps*_adjusted_ < .001) except for the pair between the involuntary and control sound conditions (*p*_adjusted_ = .063) for young adults: the time compression was stronger in the voluntary condition than in the involuntary and control sound conditions. For older adults, there were significant differences in all pairs (*ps*_adjusted_ < .001) except for the pair between the voluntary and involuntary conditions (*p*_adjusted_ = .890): the time compression was stronger in the voluntary and involuntary conditions than in the control sound condition.

***Time shift of action*.** The BM tests revealed no differences in the time shift of action between young and older adults in any preceding event conditions (voluntary: *B* = 0.67, *df* = 37.99, *p *=* *.509, *PS* = .560; involuntary: *B* = 0.57, *df* = 37.42, *p *=* *.574, *PS* = .551; control sound: *B* = 1.95, *df* = 27.69, *p *=* *.061, *PS* = .671). As for the effect of the preceding event, it was significant in both young and older adults (young: χ^2^(2) = 11.42, *p*_adjusted_* *= .007; older: χ^2^(2) = 8.96, *p*_adjusted_* *= .023). In both young and older adults, the multiple comparisons revealed that the action binding was stronger in the voluntary condition than the sound condition (young: *p* = .001; older: *p*_adjusted_ = .002), while there were no differences in the other pairs (young: *ps*_adjusted_ > .128; older: *ps*_adjusted_ > .188).

***Time shift of following sound.*** The BM tests revealed that the effect binding in the involuntary condition was stronger in the older than young adults (*B* = 4.25, *df* = 40.89, *p* < .001, *PS* = .795), but no differences in the other preceding event conditions (voluntary: *B =* 1.00, *df* = 39.81, *p* = .325, *PS* = .588; control sound: *B =* 0.67, *df* = 41.02, *p* = .504, *PS* = .560) were noticed. As for the effect of the preceding event, it was significant in older adults, χ^2^(2) = 23.19, *p*_adjusted_ < .001, and marginally significant in young adults, young: χ^2^(2) = 6.71, *p*_adjusted_* *= .070. In young adults, the multiple comparisons revealed that the effect binding was stronger in the voluntary condition than the sound condition (*p*_adjusted_ < .001), while there were no differences in the other pairs (*ps*_adjusted_ > .141). For older adults, there were significant differences in all pairs (*ps*_adjusted_ < .002) except for the pair between the voluntary and involuntary conditions (*p*_adjusted_ = .451): the effect binding was stronger in the voluntary and involuntary conditions than in the control sound condition.

**Figure 4.**
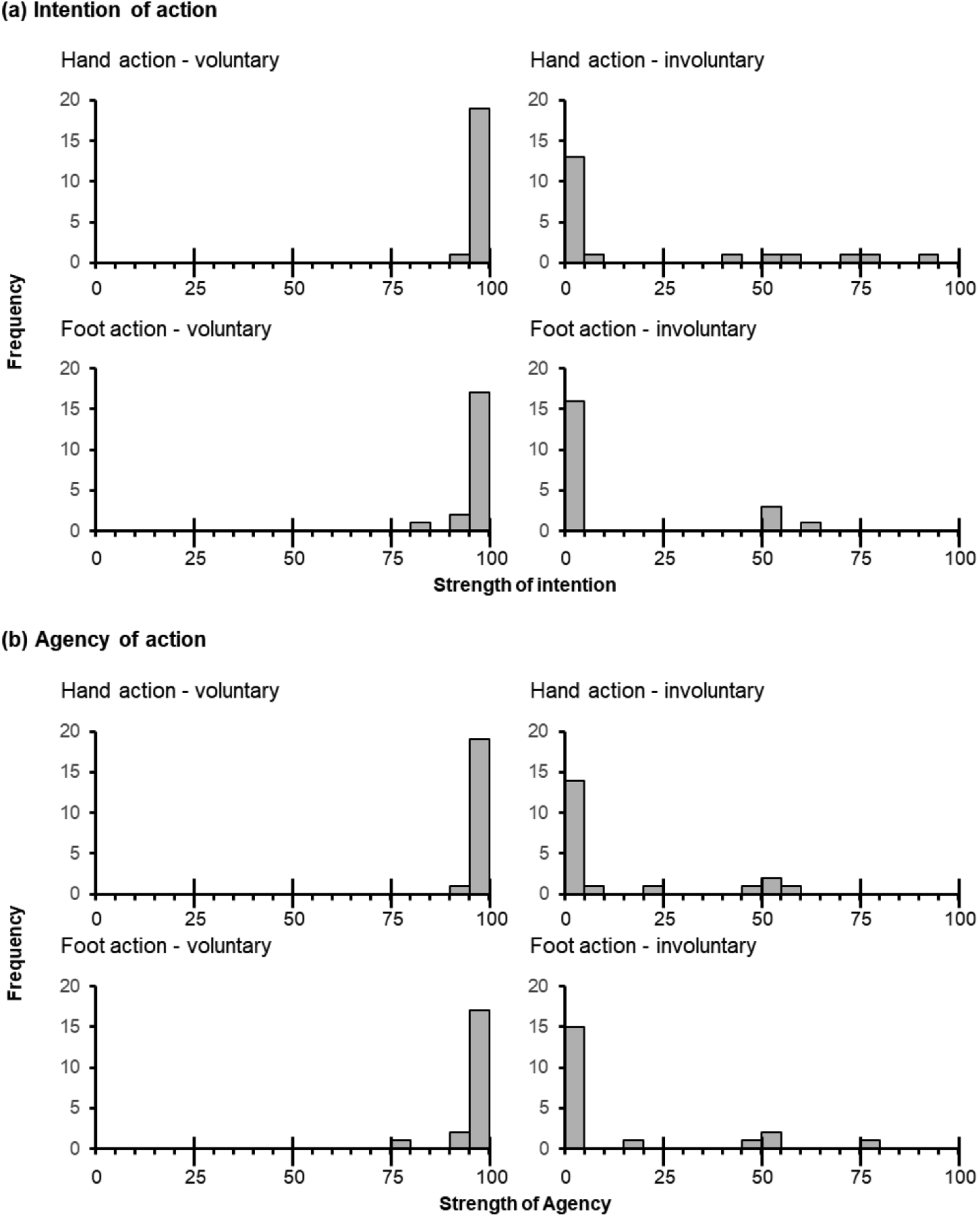
Histograms of the results of the supplemental session. (a) Results of the intention of action. (b) Results of agency of action. A larger number in the horizontal axis indicates stronger intention and agency.

***Association between time compression and sense of intention or agency.*** Figure 4 illustrates histograms of the results. Most participants felt very strong intention and agency for voluntary action and hardly perceived intention or agency for involuntary action. Correlation analyses revealed that time compression in the involuntary action condition of older adults was not significantly correlated with intention or agency strength (intensity: *ρ* = −.132, *p* = .580; agency: *ρ* = −.038, *p* = .874). These results suggest that the time compression observed in older adults cannot be accounted for by the sense of intention or agency of action.

### Foot

***Time compression.*** Figure 2(right) illustrates the results of time compression. Supplemental Table S1 shows the time shift of each condition, and Figure 3(bottom two panels) illustrates an overview of each time shift after the correction with a single event condition. The BM tests revealed that the time compression was stronger in older than young participants in the involuntary condition (*B =* 3.61, *df* = 39.69, *p* < .001, *PS* = .760), while there was no difference in the voluntary condition (*B =* 0.35, *df* = 41.94, *p* = .728, *PS* = .532) or in control sound conditions (*B =* 1.45, *df* = 42.00, *p* = .154, *PS* = .623). As for the effect of the preceding event, it was significant in young adults, χ^2^(2) = 21.29, *p*_adjusted_ < .001, and marginally significant in older adults, older: χ^2^(2) = 6.74, *p*_adjusted_ = .069. The multiple comparisons revealed significant differences in all pairs (*ps*_adjusted_ < .002) except for the pair between the involuntary and control sound conditions (*p*_adjusted_ = .275) for young adults: the time compression was stronger in the voluntary condition than in the involuntary and control sound conditions. For older adults, there were significant differences in all pairs (*ps*_adjusted_ < .011) except for the pair of voluntary and involuntary conditions (*p*_adjusted_ = .916): the time compression was stronger in the voluntary and involuntary conditions than in the control sound condition.

***Time shift of action*.** The BM tests revealed no differences in the time shift of action between young and older adults in any preceding event conditions (voluntary: *B =* 0.86, *df* = 37.22, *p *=* *.393, *PS* = .577; involuntary: *B =* 1.40, *df* = 35.49, *p *=* *.171, *PS* = .623; control sound: *B =* 2.34, *df* = 35.80, *p *=* *.025, *PS* = .693). As for the effect of the preceding event, it was significant only in young adults, young: χ^2^(2) = 13.18, *p*_adjusted_ = .003; older: χ^2^(2) = 0.67, *p*_adjusted_* *= 1.00. The multiple comparisons revealed that the action binding was stronger in the voluntary condition than the other conditions in young adults (voluntary vs. involuntary: *p*_adjusted_ = .021; voluntary vs. control: *p*_adjusted_ = .016; involuntary vs. control: *p*_adjusted_ = .994).

***Time shift of following sound*.** The BM tests revealed that the effect binding in the involuntary condition was stronger in the older than young adults (*B =* 3.19, *df* = 35.69, *p* = .003, *PS* = .741), but no differences in the other preceding event conditions (voluntary: *B =* 0.31, *df* = 41.06, *p *=* *.762, *PS* = .527; control sound: *B =* 0.04, *df* = 35.34, *p *=* *.972, *PS* = .503). As for the effect of the preceding event, it was significant in both young and older adults, young: χ^2^(2) = 16.94, *p*_adjusted_ < .001; older: χ^2^(2) = 16.22, *p*_adjusted_ < .001. In young adults, the multiple comparisons revealed that the effect binding was stronger in the voluntary condition than the other conditions (voluntary vs. involuntary: *p*_adjusted_ = .047; voluntary vs. control: *p*_adjusted_ < .001; involuntary vs. control: *p*_adjusted_ = .230). For older adults, there were significant differences in all pairs (*ps*_adjusted_ < .001) except for the pair of voluntary and involuntary conditions (*p*_adjusted_ = .909): the effect binding was stronger in the voluntary and involuntary conditions than in the control sound condition.

***Association between time compression and sense of intention or agency*.** Figure 4 illustrates histograms of the results. Most participants felt very strong intention and agency for voluntary action and hardly perceived intention or agency for involuntary action. The correlation analyses revealed that the time compression in the involuntary action condition of the older adults was not significantly correlated with intention or agency strengths for the hand (intensity: *ρ* = −.132, *p* = .580; agency: *ρ* = −.038, *p* = .874) or foot (intensity: *ρ* = .214, *p* = .366; agency: *ρ* = .161, *p* = .499). These results suggest that the time compression observed in older adults cannot be accounted for by the sense of intention or agency of action.

## General Discussion

This study aimed to investigate age-related changes in TB and their dependency on body parts using the traditional Libet clock paradigm. The results highlight marked differences in cause–effect matching between young and older participants. In young participants, the voluntary condition induced stronger time compression than the involuntary condition (and control sound condition). In contrast, in older participants, the involuntary condition induced strong time compression compared to the voluntary condition, whose time compression was comparable to the voluntary condition in young participants. Furthermore, age-related differences were observed irrespective of the action body part and were not fully explained by the strength of intention and agency of action that older participants felt in the involuntary condition.

The results of effect binding (i.e., perceptual time shift for effect event) were very similar to the results of the time compression, irrespective of the action body part, except for some statistically significant differences between preceding event conditions. That is, effect binding was significantly stronger in older than young adults only in the involuntary condition and comparable in the other conditions. The results of action binding (i.e., perceptual time shift for action event) were indistinct and seem not to be similar to the time compression. Additionally, action binding was also consistent between action body parts in young adults, but not clear in older adults. In young adults, both hand and foot action induced significant influences of the preceding events. In older adults, however, only hand action induced significant influence of the preceding events.

### Validity of Our Experimental Setting and (Almost No) Influence of Body Part

Voluntary hand action induced significant time compression in young participants, similar to previous studies, including Haggard et al. (2002). Involuntary hand action also induced significant time compression, although it was significantly weaker than voluntary action. This was not observed in Haggard et al. (2002). However, it was occasionally reported when involuntary action was induced by mechanical devices (Buehner, 2015; Cavazzana et al., 2014, 2017). These results indicated that our experimental setting was appropriate to investigate the perceptual time interval between an action and the following event.

One of the novel findings in this study is that time compression was similarly induced both in the hand and foot conditions, although the action binding was slightly different. The results suggest that time compression and effect binding were not influenced by the body part of the action in either participant group. This indicates that various differences between hand and foot, such as functional differences (e.g., the hand can grasp something; the foot can support whole body weight, jump, and run), anatomical differences (e.g., the difference in neurotransmission length), and a difference in aging (rapid decreasing of lower limbs’ muscle), have no impact on time compression and action/effect binding. This suggests that relatively higher-order mechanisms or general motor control of action rather than motor control specific to a local body part are involved in these time perceptions. Numata et al. (2022) revealed that both finger and foot actions can synchronize with periodic tones accurately, although neurotransmission lengths differed. That is, the perceptual timing of the limb's action is calibrated while considering the difference in neurotransmission lengths. Our results that hand and foot action induces similar TBs suggest that the calibration mechanism works also for the process of TB.

Regarding the small differences in action binding between hand and foot only in older participants, this might be due to the unfamiliarity or unnaturality of the foot action, given that the results of the hand action are almost consistent with those of time compression or the effect binding. It might be possible that older participants who have less flexibility in foot action were more influenced by the unfamiliarity or the unnaturality. It must be noted that any differences in the results of the body parts should be interpreted cautiously because the effects of condition order might be confounded.

### Age-Related Changes in Time Compression

Older participants in this study exhibited significant time compression in the voluntary action conditions, which was comparable to that of young participants. This is inconsistent with the results of Cavazzana et al. (2017), where older participants and children exhibited lesser time compression than young participants. The reason put forth by them was that children and older adults have difficulty inhibiting their attention on voluntary action and moving to the following sound. The attentional focus left on the voluntary action may reduce the influence of the following sound, resulting in reduced action binding. Consequently, total time compression was weakened. One of the main differences between Cavazzana et al. (2017) and ours is the experimental method: Cavazzana et al. (2017) used rapid serial presentation of alphabets, which was developed by the authors (Cavazzana et al., 2014, 2017), whereas we used the conventional Libet clock method. Lorimer et al. (2020) suggested that Cavazzana et al.'s (2014, 2017) method may be too cognitively demanding for children, and suggested the possibility that TB can be observed in children in a paradigm that does not place excessive demands on attentional resources. Indeed, Lorimer et al. (2020) used a less demanding method they developed to show that time compression was observable in children at least (see also Blakey et al., 2019). Some methodological differences between these studies such as higher familiarity and followability of a clock hand than a rapid serial presentation of alphabets might reduce cognitive demands. Furthermore, also in older adults, the rapid presentation task may require more cognitive resources than younger adults because the rapid presentation requires rapid processing of judgment and attentional control. Some studies using sequential presentation showed that sensitivity to a temporal order of stimuli presentation degraded in older adults (de Boer-Schellekens & Vroomen, 2014; Ulbrich et al., 2009), and that older adults exhibited longer attentional blink (Lahar et al., 2001; Langley et al., 2008; Maciokas & Crognale, 2003) and a disadvantage in attention inhibition (Hasher et al., 1999). The higher cognitive load in Cavazzana's method may also reduce TB in older adults.

Another novel finding of our study is that not only voluntary action but also involuntary action induced significant time compression comparable to voluntary action. Additionally, our results showed that the time compression of involuntary action was induced independently of the participant's intention or sense of agency of action. These suggest that our data cannot be fully explained by the traditional view of TB that intention or sense of agency plays a critical part in TB (Haggard et al., 2002). Several recent studies have demonstrated that time compression can be induced without the intention of action (Buehner, 2012; Lorimer et al., 2020; Suzuki et al., 2019). These studies argue that an essentially important factor of the binding phenomenon is causality between action and effect rather than the intention of action, although the intention can influence the causality. According to the idea of causality, the time compression of involuntary action in our study may reflect some age-related changes in cognition of causality between action and effect. Desantis et al. (2011) revealed that causal belief between action and effect influences TB, especially effect binding rather than action binding. This is consistent with our results of Experiment 1 that the main part of the time compression of involuntary action was the results of the effect binding instead of the action binding. Mutter et al. (2007) reported that prior beliefs strongly influence older participants’ contingency judgments, whereas young participants appropriately integrate prior beliefs with concurrent information about a given event. According to this idea, in our experiment, repeated exposures to the contingency between finger (foot) movements (triggered by own intention or a machine) and effect might have the ability to form prior knowledge of the causality, and older participants may be more strongly influenced regarding event time estimations than young participants. The time compression in young adults’ involuntary condition observed in this and previous studies (Buehner, 2015; Cavazzana et al., 2014, 2017) may reflect the influence of prior knowledge formed by repeated presentation of mechanical button press and its consequence. The prior knowledge indicates clear causality between action and effect, and it is independent of an agent of a button press (machine or participant). The causality binds two events (button press and delayed sound) like a single event and makes a strong bias of time compressions equally in both voluntary and involuntary actions.

The prior knowledge of causality itself does not seem different between young and older participants who have normal cognitive ability in our experiment. However, young participants have more sensitive modalities (somatic sensation for button press and auditory sensation for delayed sound) than older participants, and the sensitive modalities indicate that the button press and delayed sound happen at different timings. Specifically, in the involuntary condition of young participants, this conflict between the prior causality and sensory information might make the time compression weaker. In contrast, in the voluntary condition, an intention signal is provided. The sensory information would be strongly suppressed by the motor system (“sensory attenuation”; Blakemore et al., 1998). Therefore, the prior causality would hardly conflict with the sensory information, and, consequently, stronger time compression is induced than involuntary action. Contrarily, in older participants, the reliability of sensory information (especially somatic sensation) degrades (Seidler et al., 2010). Therefore, the prior knowledge of causality, which has relatively high reliability, leads to strong time compression, especially in involuntary action, where no obvious intention signal exists. Further, evidence shows that the width of the temporal window of multisensory integration increases with advancing age (e.g., Bedard & Barnett-Cowan, 2016). This might also help the brain to make an inference that multisensory signals (visual and somatosensory signals, and the following auditory signal in this study) are caused by a single external event. This is consistent with previous studies on TB, which suggest that perceptual grouping of multisensory information from action and effect is significantly related to time compression (Kawabe et al., 2013; Klaffehn et al., 2021; Lush et al., 2019; Yamamoto, 2020). Further, the idea that both self and mechanical actions induce a strong bias of time compression based on the causality is also consistent with the results of the subjective rating, wherein older participants recognized reasonable intention and agency in both voluntary and involuntary action. At least, these results indicate that intention and agency are not essential factors of time compression in older participants.

This study has some limitations. First, young adults did not participate in the supplementary session where the degrees of sense of intention and agency of action were explicitly asked. Few older participants reported an explicit sense of intention and agency of action in the involuntary condition, although these minor opinions were captured in the majority by the statistical analysis. It was not clear whether these reports of a sense of intention and agency in involuntary action were specific to older participants. Although we concluded that explicit sense of action intention and agency did not influence time compression for involuntary action, age-related changes in the explicit sense of intention and agency might be involved in perceived time compression. Second, we additionally measured explicit sense of intention and agency in the supplementary session, but the TBs were not measured at the same time. Imaizumi and Tanno (2019) suggest that the correlation between TB and an explicit sense of agency might not be observable if they were measured in different sessions. Thus, this separated measurement might reduce the correlation between them. Third, the experiment was not counterbalanced between hand and foot actions. The results showed that both hand and foot actions induced similar time compression. Therefore, the order effect may have confounded some influence of the body parts. Finally, older participants performed only 10 repetitions for each condition while young participants performed 20 repetitions. Therefore, this difference and the difference in total experiment time might influence the results, and make a strict comparison between the participant groups difficult. Therefore, TB by voluntary action might be different between the participant groups, even though our results showed very similar TB. These should be properly controlled for in future studies. However, the number of trials may not have a strong influence on TB, at least in young adults, because numerous TB studies have repeatedly shown a difference in time compression between voluntary and involuntary conditions (Haggard, 2017; Haggard & Chambon, 2012; Haggard et al., 2002; Moore & Obhi, 2012), but no previous study has reported an influence of the number of trials.

### Conclusion

This study investigated age-related changes in TB. The results indicated that TB was induced in older participants, and it was comparable to that in young participants. Moreover, only in older participants, not only voluntary action but also involuntary action induced the time compression. These phenomena can be the outcomes of age-related change in cognition of causality between action and effect.

## Supplemental Material

sj-docx-1-ipe-10.1177_20416695231208547 - Supplemental material for Age-related differences in temporal binding and the influence of action body parts Click here for additional data file. Supplemental material, sj-docx-1-ipe-10.1177_20416695231208547 for Age-related differences in temporal binding and the influence of action body parts by Yoshitaka Fujii, Naoki Kuroda, Ryo Teraoka, Shinya Harada, and Wataru Teramoto in i-Perception
