# Supplementary material for: Age-related differences in temporal binding and the influence of action body parts
Source: Iperception. 2023 Oct 30;14(5):20416695231208547. doi: 10.1177/20416695231208547 (PMC10617286; doi:10.1177/20416695231208547)
Supplement: sj-docx-1-ipe-10.1177_20416695231208547 - Supplemental material for Age-related differences in temporal binding and the influence of action body parts [file sj-docx-1-ipe-10.1177_20416695231208547.docx]

**Supplementary Table 1:** Mean time-shift across all participants.


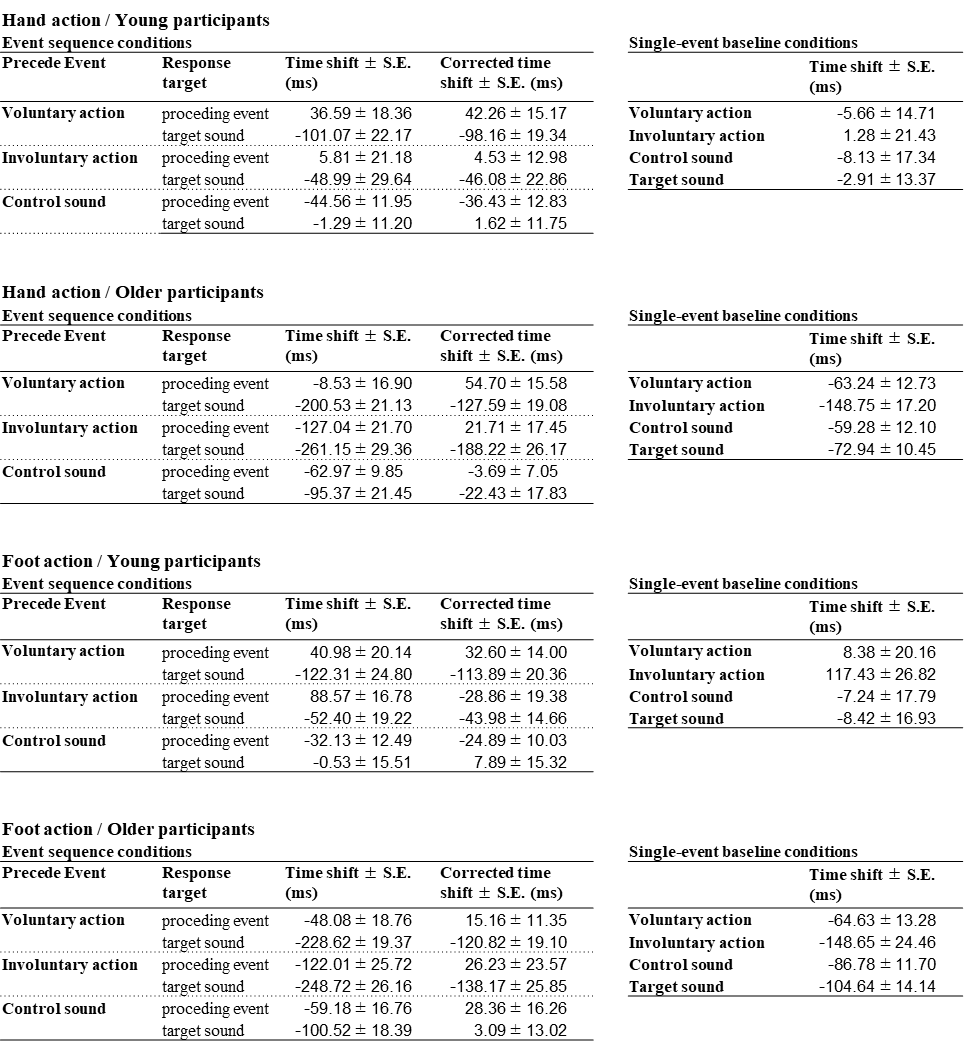


Positive values of the time shifts indicate perceptual delay. Corrected time shift means the time shift that is corrected by the corresponding results of the single-event baseline condition. *S.E.* means standard error.
